# Systematic reviews in surgery—recommendations from the Study Center of the German Society of Surgery

**DOI:** 10.1007/s00423-021-02204-x

**Published:** 2021-06-15

**Authors:** Eva Kalkum, Rosa Klotz, Svenja Seide, Felix J. Hüttner, Karl-Friedrich Kowalewski, Felix Nickel, Elias Khajeh, Phillip Knebel, Markus K. Diener, Pascal Probst

**Affiliations:** 1 Study Center of the German Society of Surgery (SDGC), University of Heidelberg, Im Neuenheimer Feld 130.3, 69120 Heidelberg, Germany; 2 Department of General, Visceral and Transplantation Surgery, University of Heidelberg, Im Neuenheimer Feld 420, 69120 Heidelberg, Germany; 3 Institute of Medical Biometry and Informatics, University of Heidelberg, Im Neuenheimer Feld 130.3, 69120 Heidelberg, Germany; 4 Department of General and Visceral Surgery, University Hospital Ulm, Albert-Einstein-Allee 23, 89081 Ulm, Germany; 5 Department of Urology and Urological Surgery, University Medical Center Mannheim, University of Heidelberg, Theodor-Kutzer-Ufer 1-3, 68167 Mannheim, Germany

**Keywords:** Synoptic evidence, Systematic review, Surgery, Meta-analysis, Evidence-based medicine

## Abstract

**Background:**

Systematic reviews are an important tool of evidence-based surgery. Surgical systematic reviews and trials, however, require a special methodological approach.

**Purpose:**

This article provides recommendations for conducting state-of-the-art systematic reviews in surgery with or without meta-analysis.

**Conclusions:**

For systematic reviews in surgery, MEDLINE (via PubMed), Web of Science, and Cochrane Central Register of Controlled Trials (CENTRAL) should be searched. Critical appraisal is at the core of every surgical systematic review, with information on blinding, industry involvement, surgical experience, and standardisation of surgical technique holding special importance. Due to clinical heterogeneity among surgical trials, the random-effects model should be used as a default. In the experience of the Study Center of the German Society of Surgery, adherence to these recommendations yields high-quality surgical systematic reviews.

## Background

Systematic reviews (SRs) are of high importance for decision-makers in the healthcare system and crucial to the development of clinical guidelines. SRs connect the results of single studies on the same topic, thereby providing clinicians with the best foundation for evidence-based treatment of their patients. Additionally, SRs can identify research gaps and provide recommendations for future clinical trials in terms of effect estimates and meaningful endpoints.

Generally speaking, a systematic review has five steps: *formulating* the research question; *identifying*, *selecting*, and *assessing* the relevant literature; and *synthesis*, i.e. interpretation of quantitative results (such as by meta-analysis) in light of the quality of the studies included [1]. SRs are considered original research in most journals, especially when a meta-analysis is performed [2, 3].

Only a limited number of surgical interventions are based on randomised controlled trials (RCTs) [4, 5] representing the highest level of evidence [6]. Therefore, surgical SRs are especially at risk of the falling prey to the classic ‘garbage in, garbage out’ problem [7]. To avoid this designation, SRs in the field of surgery in particular must address the quality of all included trials, paying special attention to specifics about surgical trial methodology. Otherwise, poor-quality SRs run the risk of encouraging poor treatment decisions and incurring unnecessary costs within the healthcare system [8].

In 2005, the Study Center of the German Society of Surgery (www.sdgc.de) founded a systematic review working group. This working group is committed to disseminating the know-how required to plan, conduct, and publish SRs among German surgeons and to aid them throughout this process. Since 2005, the group published more than 70 SRs and specific literature was created on the methodology of surgical SRs [9–12]. In this article, recommendations for conducting a state-of-the-art surgical SR with or without meta-analysis are provided.

## Recommendations

Recommendations are given for each step of an SR. General recommendations are followed by specific recommendations important to surgical reviews.

### Formulating the research question

At the beginning of an SR, it is essential to clearly state the research question. The aim of an SR should be to answer an unanswered and important clinical question without an existing SR or for which new primary evidence has become available. Therefore, before beginning an SR, registers and literature databases should be screened for existing SRs on the subject.

**Fig. 1:**
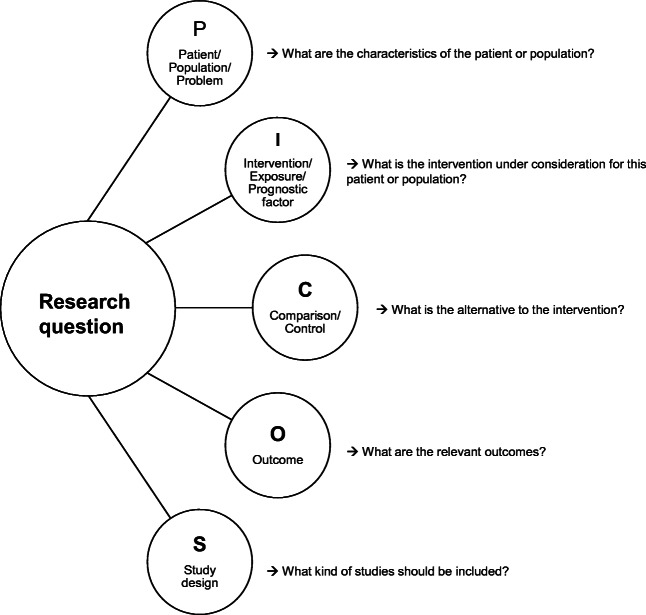
PICOS criteria

To define a well-focused and answerable research question, PICOS criteria [13, 14] should be used. PICOS stands for Patient, Intervention, Comparison, Outcome, and Study design (Fig. 1). In surgical reviews, the following specific characteristics should be clarified:

Patient:

- Will only adult patients be included?
- Will only patients with a malignant disease be considered?
- Will patients with previous surgery be excluded?
- Will patients with neoadjuvant therapy be excluded?
- etc.

Intervention:

- Due to the complexity of surgical interventions, it is important to define the exact procedure or group of relevant procedures to guarantee comparability and to ensure that if interventions are deemed effective, they can actually be reproduced and implemented in clinical practice.
- In so doing, the recommendations of Blencowe et al. [15] should be followed. Their paper describes a framework for deconstructing surgical interventions into their constituent components and provides steps to clarify details of the intervention under evaluation (e.g. concerning the intervention ‘robotic partial pancreatoduodenectomy’; this list could include positioning of the robot, incision and access, dissection, reconstruction, anastomoses, and closure).

Comparison: adequate controls in surgical reviews can be any of the following, depending on the research question:

- One or more state-of-the-art surgical intervention(s) (‘gold standard’, e.g. intervention: laparoscopic pancreatoduodenectomy versus comparison: open pancreatoduodenectomy [16])
- Conservative treatment without surgical intervention such as the administration of a drug (e.g. intervention: metabolic surgery versus comparison: medical treatment in patients with type 2 diabetes [17])
- Surgical placebo [18, 19] (e.g. intervention: arthroscopic debridement of the knee versus comparison: sham surgery, i.e. skin incision only in patients with osteoarthritis [20])

Outcome: a main outcome should be defined and clear definitions should be applied to it. Common outcomes in surgical reviews include the following:

- Intraoperative outcomes, such as operative time or blood loss
- Perioperative morbidity and mortality and specific postoperative complications
- Patient-reported outcome measures, such as functional outcomes and quality of life
- Long-term oncological or functional outcomes
- Economic evidence, such as cost-effectiveness

Study design: depending on the available literature, it has to be decided what study type(s) should be included:

- RCTs
- Non-randomised prospective (comparative) studies
- Cohort-type studies
- Case series

After formulating the research question, a research protocol should be developed. It is recommended to register the SR in a public register, e.g. PROSPERO [21, 22], and/or to publish the protocol in a peer-reviewed journal. Registration improves the quality of performance and the transparency in an SR, prevents duplicate work, and reduces the risk of selective reporting by providing an a priori analysis plan. The PRISMA-P checklist should be followed when writing an SR protocol [23].

### Identifying potentially relevant literature

The aim of a systematic literature search is to identify all relevant studies regarding the research question without including any irrelevant studies at the same time. However, this is impossible: a too-narrow or highly specific search strategy will miss relevant studies, whereas a too-broad search strategy would require screening thousands of irrelevant studies.

It is recommended to start with a wide initial scoping search and to adjust the search accordingly. Depending on the number and content of studies retrieved, the literature search should then be modified. It is important to include all free text synonyms of a term, e.g. ‘Whipple’, ‘pancreatoduodenectomy’, ‘pancreaticoduodenectomy’, ‘resection of the pancreatic head’, and ‘pancreatic head surgery’. Additionally, using medical subject headings (MeSH) improves the quality of the literature search. If there are still too many hits, adjuvant filters, such as a time window (e.g. time of the first available robotic surgery) or study type (e.g. RCT filter), can be considered. After the initial search, terms can be revised according to whether or not relevant, previously known literature can be found using these search terms.

An SR should always search more than one database. The following databases are recommended for surgical SRs: MEDLINE (via PubMed), Web of Science, and Cochrane Central Register of Controlled Trials (CENTRAL). For surgical topics, MEDLINE has the highest recall (92.6%) and precision for non-randomised studies (NRS, 5.2%), whereas CENTRAL is more sensitive (88.4%) and has the highest precision (8.3%) for RCTs. The combination of MEDLINE and CENTRAL has a 98.6% recall for RCTs. For NRS, the highest recall (99.5%) is retrieved by the combination of MEDLINE and Web of Science [9]. EMBASE does not contribute substantially to reviews on a surgical intervention. However, for research questions involving a drug intervention (e.g. medical vs. surgical intervention), the inclusion of EMBASE should be considered [9]. An additional hand search is also recommended; reference lists of relevant articles found by the literature search should be screened for further relevant articles not found by the literature search. It is also advisable to seek professional assistance by a librarian if the research team has limited experience in conducting a search.

### Selecting the relevant literature

The most relevant inclusion and exclusion criteria for study selection have already been outlined by PICOS. However, these a priori stated criteria could sometimes be altered depending on the number of eligible studies. Besides the clinically based inclusion and exclusion criteria, the authors must determine which study designs should be included. Although the quantity and quality of surgical RCTs have increased in some specialities [24], there is still a general lack of high-quality trials in many areas of surgery. Therefore, the aim of an SR should be to find the best available evidence. If four or more RCTs are available, then NRS can be omitted.

Limiting oneself to articles in English should be avoided and endeavours should be made to translate articles in other languages. If non-English articles are excluded, the exact number of those articles should be provided.

Overall, the final eligibility criteria should be stated clearly, with the publication of results. While one reviewer can conduct the initial screening of titles and abstracts, it is recommended that both initial screening and full-text screening (according to eligibility criteria) be completed by two researchers [13]. Any disagreement during the screening process should be resolved by consensus, or by consultation with a third reviewer. The selection process should be documented with a PRISMA flowchart ( http://www.prisma-statement.org/documents/PRISMA_2020_flow_diagram_new_SRs_v2.docx) [22]. In addition, during full-text screening, the reason for exclusion should be given.

### Assessing the quality of primary studies

Studies meeting all inclusion criteria enter the next stage of data extraction. This step should be performed using a standardised form (electronic or paper-based), which was piloted in initial trials and revised accordingly. Besides the extraction of the relevant endpoints, some descriptive details should be presented in a tabular form in the publication, e.g. author, title, year and geographical origin of publication, number of participants, and specific information on the surgery performed. Additionally, baseline information such as tumour stage or performance status could be included when some specialised centres perform surgery on more complex cases, thus potentially impacting the outcome. It is advisable to involve a statistician at this stage, in order for data to be extracted in a manner which allows for the calculations planned.

Outcomes should be predefined before screening the eligible literature and should refer to PICOS. A main outcome, such as the primary endpoint in a trial, should be clearly defined. For easier comparison among outcomes, clearly defined endpoints should be used whenever possible. For example, postoperative complications should be evaluated according to the Clavien-Dindo classification [25] and endpoints like postoperative pancreatic fistula should be extracted according to ISGPS definitions [26]. The use of differing definitions among the studies included should be mentioned, and their possible impact on quantitative analysis should be discussed (and could be assessed) in a sensitivity analysis.

Assessing a study’s methodological quality is a key aspect of every well-performed SR. Only in light of methodological quality can the quantitative merit of a study be interpreted. This aspect is even more important for surgical SRs, due to the lack of standardisation in surgical trials compared to pharmacological trials. Critical appraisal of the studies with a validated tool is therefore also recommended. Risk of bias should be reported in a paragraph dedicated to it in the results section. Different risk of bias assessment tools exists for different study types (see Table 1 for recommendations).

**Table 1:** Risk of bias assessment tools

Tool	Study type
RoB 2.0 [27]	RCTs
ROBINS-I [28]	NRS (clinical controlled trial, case-control)
MINORS [29]	NRS
Downs and Black [30]	NRS (case series)
QUIPS [31]	Prognostic studies

Some other tools which have been critically dealt with, especially for reporting bias, exist for different types of studies [32].

The critical appraisal of surgical trials has many specifics that should be addressed, including but not limited to blinding, industry bias, experience, and standardisation of intervention.

Generally, in RCTs, blinding is favourable to reduce detection and performance bias. In surgical trials, blinding is not easy to apply [33, 34] and not every lack of blinding will lead to performance or detection bias. The usual term ‘double-blind trial’ is not reasonably transferable to surgical trials and should be avoided. Therefore, it is recommended to report specifically which (if any) study contributor (whether patient, surgeon, data collector, outcome assessor, or data analyst) was blinded.

Specifically, for surgical trials, it has been shown that industry funding leads to more positive results than independent funding [11]. Therefore, in surgical trials, which investigate an intervention with an inherent industrial interest, the source of funding should be extracted and subgroup analysis of industry-funded and non-industry-funded trials should be performed [35].

Furthermore, experience of the operating surgeon(s) and possible learning curves should be addressed, since these can specifically influence comparisons between established and new surgical interventions [36, 37]. Validated surgical quality control measures should be implemented since significant bias can otherwise result [16, 38].

### Small-study effects and publication bias

Small-study effects describe the phenomenon that smaller studies sometimes show different treatment effects than larger ones [39]. The most well-known (albeit not the only) reason for this phenomenon is the presence of publication bias in the data. This bias occurs if the chance of a smaller study being published increases when it shows a stronger treatment effect, which in turn biases the results of the meta-analysis and the SR [40]. Small-study effects can be graphically illustrated by a funnel plot [41], where estimated treatment effects are shown against a measure of their precision. In the absence of small-study effects, the funnel plot shows a symmetric scattering of the treatment effects around their average in the form of a triangle, with more variation in smaller (imprecise) studies than in larger (precise) ones. There are several well-known statistical tests for measuring small-study effects and asymmetry in funnel plots, e.g. the non-parametric Begg and Mazumdar test [42] or the parametric Egger regression test [43]. However, the power of statistical tests is known to be low, and interpretability of funnel plots is often limited, due to the low number of studies contributing to the analysis and should therefore only be used when more than ten studies are available [44].

Finally, surgical interventions are complex and comprise multiple components that might be accompanied by concomitant interventions such as anaesthesia and perioperative management [45]. Also, different levels of experience among operating surgeons as well as case volumes among the hospitals where a trial was performed might influence outcomes. For example, an SR addressing how details about surgical interventions are reported found a clear standardisation of the intervention in less than 30% of the included RCTs [46]. Also, measurements of adherence to the intervention might be missing. Consequently, the level of standardisation of the intervention in the included trials needs to be reported and in case of missing standardisation in some trials, a subgroup analysis including only trials containing clear reporting of the performed intervention might be necessary.

Contrary to screening, data extraction and risk of bias assessment should be performed by at least two researchers [13] and any differences among them should be resolved by consensus, or by consultation with a third reviewer.

Furthermore, whether or not any measures of surgical experience were gathered in the primary trials, as well as its impact on outcome, should also be assessed in a sensitivity analysis if such information is available.

The results of the critical appraisal should be clearly described in the final report of any SR, and the impact of the quality of evidence on interpretation of the results should be discussed. For this reason, simply providing a summary score of quality assessment on the study level is strongly discouraged.

### Synthesis of quantitative and qualitative results

The last step of an SR is to synthesise the extracted data, i.e. quantitative analysis (meta-analysis), and merge it with qualitative analysis (critical appraisal/risk of bias).

A quantitative analysis should always be evaluated critically, and determining whether or not a meta-analysis makes sense should be based on the extracted data. A meta-analysis is justified if sufficiently homogeneous studies can be assimilated for statistical analysis. Different effect measures are used to summarise treatment effects depending on the scale of the outcome. Commonly used effect measures are the risk difference (RD), the risk ratio (RR) and the odds ratio (OR) for binary outcomes, the mean difference (MD) or standardised mean difference (SMD) for continuous outcomes, and hazard ratios (HR) for time-to-event outcomes [47]. When choosing adequate effect measures for the included endpoints, statistical aspects, as well as convention or interpretability, need to be considered [48].

A meta-analysis summarises the results of individual studies as a combined effect estimate. As studies usually differ in the number of patients included and therefore vary in their precision, the naïve approach of simply averaging the effects across studies is not recommended. Instead, weighted effect estimators that use the precision of the identified studies are commonly used to include the individual studies according to their precision and more weight will be assigned to large, precise studies than to small, imprecise ones [49]. Analytical techniques can be broadly classified into two categories: the fixed-effect (now often called common-effect) model and the random-effects model. The fixed-effect model assumes that all studies would yield the same result if they were infinitely large. Statistically, this means that random error is assumed to be attributable solely to the differences that occur in patients within a study and not due to any variations among the trials [50]. As a rule, this assumption is unrealistic, as small variations in study design and surgical technique almost always occur. In the random-effects model, such between-trial heterogeneity is considered, which then increases the imprecision with which the combined effect is estimated in a random-effects meta-analysis, i.e. leading to wider confidence intervals. Therefore, the use of the random-effects model is generally recommended in surgical SRs with meta-analysis irrespective of the presence of statistical heterogeneity as explained below [51]. Different estimation approaches are available. Well-established approaches include (upon others) the method of moment estimator of DerSimonian-Laird and the restricted maximum likelihood estimator [52–54]. A typical graphical illustration of a meta-analysis is the forest plot. In a forest plot, the results of individual studies (estimated effects and confidence intervals) are displayed along with the results of the meta-analysis, the estimated between-trial heterogeneity, and the weight that is assigned to each study. It is recommended to calculate and display statistical heterogeneity by reporting between-trial variance as *τ*^2^ and the *I*^2^ as relative measure incorporating between- and within-trial variances [55, 56]. If this is unavoidable, e.g. due to a small number of included studies, the forest plot should be stratified or undergo sub-group analysis.

A variety of statistical approaches exist and determining which method is ‘best’ in a specific setting is often a matter of some debate. Therefore, the performance of sensitivity analyses to test the robustness of results is strongly advised. Commonly performed sensitivity analyses include a change of estimation method, an analysis with high-quality studies only, or an analysis including only studies with recent surgical methodology. Randomised and non-randomised studies should always be analysed separately.

Data synthesis should answer the research question by merging the qualitative and quantitative analyses. Quantitative statements about outcomes, e.g. ‘operation A is superior to operation B’, should be accompanied by the certainty of evidence. For this step, the GRADE approach is recommended and certainty of evidence should be rated as very low, low, moderate, or high [57]. Apart from risk of bias, GRADE also includes other clinical and statistical characteristics which might influence the certainty of evidence. A ‘summary of findings’ table (https://gradepro.org/) showing quantitative results alongside the certainty of evidence for each outcome is therefore recommended. Moreover, since abstracts are read more frequently than full publications, the synthesis including certainty of evidence should be part of the abstract’s conclusion. Finally, PRISMA guidelines (http://prisma-statement.org/) should be followed when preparing and reporting SR results [22].

## Summary

A thoroughly conducted SR of high-quality trials achieves the highest level of evidence. Ultimately, an SR of this calibre provides more comprehensive evidence for clinical decision-making than a single study alone. An SR follows a structured process and requires specific methodology where surgical interventions are under investigation (Box 1).

**Box 1:**
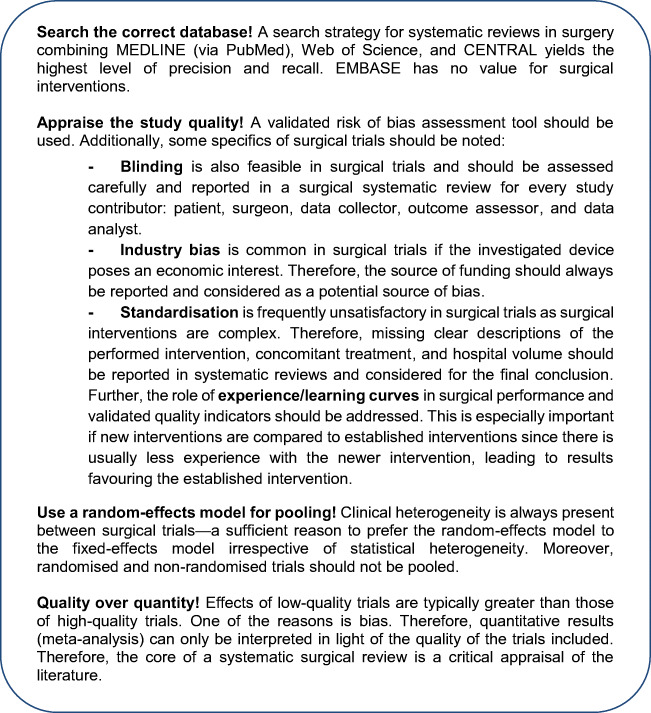
Summary of recommendations by the Study Center of the German Surgical Society for the conduct of a systematic review
